# Soufeng sanjie formula alleviates collagen-induced arthritis in mice by inhibiting Th17 cell differentiation

**DOI:** 10.1186/s13020-021-00448-9

**Published:** 2021-05-13

**Authors:** Di Hua, Jie Yang, Qinghai Meng, Yuanyuan Ling, Qin Wei, Zhigang Wang, Qingyun Wei, Jiao Chen, Juan Ye, Xuan Han, Kelei Su, Weikang Kong, Chao Xu, Peng Cao, Chunping Hu

**Affiliations:** 1 Affiliated Hospital of Integrated Traditional Chinese and Western Medicine, Nanjing University of Chinese Medicine, Nanjing, 210028 China; 2 College of Pharmacy, Nanjing University of Chinese Medicine, Nanjing, 210023 China; 3 Affiliated Hospital of Yifu, Nanjing Medical University, Nanjing, 211166 China

**Keywords:** Collagen-induced arthritis, Soufeng sanjie formula, Th17, Th17/Treg, RORγt

## Abstract

**Background:**

Rheumatoid arthritis (RA) is a chronic autoimmune disease. Soufeng sanjie formula (SF), which is composed of scolopendra (dried body of *Scolopendra subspinipes mutilans* L. Koch), scorpion (dried body of *Buthus martensii* Karsch), astragali radix (dried root of *Astragalus membranaceus* (Fisch.) Bge), and black soybean seed coats (seed coats of *Glycine max* (L.) Merr), is a traditional Chinese prescription for treating RA. However, the mechanism of SF in treating RA remains unclear. This study was aim to investigate the anti-arthritic effects of SF in a collagen-induced arthritis (CIA) mouse model and explore the mechanism by which SF alleviates arthritis in CIA mice.

**Methods:**

For in vivo studies, female DBA/1J mice were used to establish the CIA model, and either SF (183 or 550 mg/kg/day) or methotrexate (MTX, 920 mg/kg, twice/week) was orally administered to the mice from the day of arthritis onset. After administration for 30 days, degree of ankle joint destruction and serum levels of IgG and inflammatory cytokines were determined. The balance of Th17/Treg cells in the spleen and lymph nodes was analyzed using flow cytometry. Moreover, the expression levels of retinoic acid receptor-related orphan nuclear receptor (ROR) γt and phosphorylated STAT3 (pSTAT3, Tyr705) in the spleen were detected by immunohistochemistry. Furthermore, the effect of SF on Th17 cells differentiation in vitro was investigated in CD4^+^ T cells under Th17 polarization conditions.

**Results:**

SF decreased the arthritis score, ameliorated paw swelling, and reduced cartilage loss in the joint of CIA mice. In addition, SF decreased the levels of bovine collagen-specific IgG in sera of CIA mice. SF decreased the levels of inflammatory cytokines (TNF-α, IL-6, and IL-17A) and increased the level of IL-10 both in the sera and the joint of CIA mice. Moreover, SF treatment rebalanced the Th17/Treg ratio in the spleen and lymph nodes of CIA mice. SF also reduced the expression levels of ROR γt and pSTAT3 (Tyr705) in the spleen of CIA mice. In vitro, SF treatment reduced Th17 cell generation and IL-17A production and inhibited the expression of RORγt, IRF4, IL-17A, and pSTAT3 (Tyr705) under Th17 polarization conditions.

**Conclusions:**

Our results suggest that SF exhibits anti-arthritic effects and restores Th17/Treg homeostasis in CIA mice by inhibiting Th17 cell differentiation.

**Figure:**
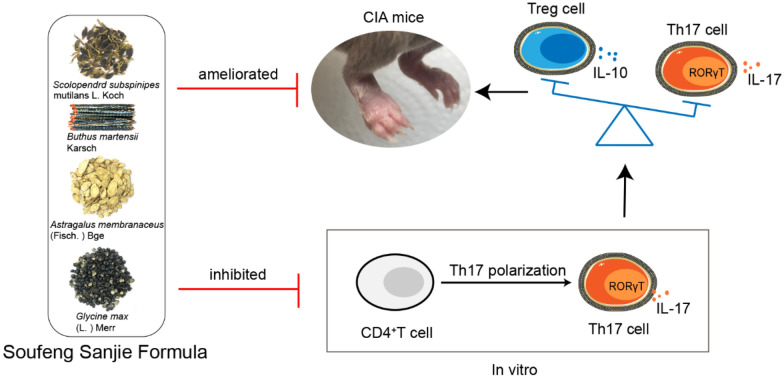

**Supplementary Information:**

The online version contains supplementary material available at 10.1186/s13020-021-00448-9.

## Background

Rheumatoid arthritis (RA) is a chronic autoimmune disease in which multiple immune cell types and signaling networks malfunction to elicit a maladaptive tissue repair process that leads to organ damage, predominantly in the joints [1]. The most prominent feature of RA is swelling and deformation of the hands, wrists, feet, and knees [2]. RA may occur at any age, and the cumulative lifetime risk of developing adult-onset RA has been estimated at roughly 3.6% for women and 1.7% for men, with a peak incidence age of 50 to 60 years [3].

Substantial evidence has emerged supporting a role for immune cells, including T cells, macrophages, and fibroblasts, as well as inflammatory cytokines, in the initiation and progression of RA [4, 5]. CD4^+^ T helper (Th) cells regulate immunity and inflammation through antigen-dependent activation and cytokine-dependent differentiation into functionally distinct effector and regulatory T cell subsets. The proinflammatory cytokine tumor necrosis factor-α (TNF-α) regulates Treg cells through Foxp3 dephosphorylation, and interleukin-6 (IL-6) and TNF-α trigger naïve CD4^+^ T cell differentiation into Th17 cells [6, 7]. However, the imbalance of regulatory T cells (Tregs) and Th17 cells can cause autoimmune diseases [8]. Th17 cells have been shown to stimulate fibroblast-like synoviocytes via interleukin-17 (IL-17) and expand synovial-resident innate lymphoid cells in inflamed joints [9]. Therefore, the inhibition of Th17 cell activity and IL-17 expression has become a potential therapeutic target for the treatment of RA.

The current drugs for the treatment of RA are mainly conventional synthetic agents (e.g., methotrexate) and targeted synthetic disease-modifying anti-rheumatic drugs (DMARDs) [10]. Several biological DMARDs, including TNF-α blockers and IL-6 receptor (IL-6R) inhibitors, have been used to treat RA [11]. However, most of these drugs act slowly or produce severe adverse reactions after long-term application. Many RA patients do not respond favorably to methotrexate, and not all RA patients respond to biological DMARDs in terms of retardation of joint destruction [12, 13]. Therefore, drugs with good curative effect and low adverse reactions are needed for RA patient.

Traditional Chinese medicine has a significant effect on treating long-term chronic diseases such as RA [14]. It has been reported that the combination of scolopendra and scorpion with herbal medicine is effective in the clinical treatment of RA [15]. Soufeng sanjie formula (SF) is thus a prescription, which is composed of scolopendra (dried body of *Scolopendra subspinipes mutilans* L. Koch), scorpion (dried body of *Buthus martensii* Karsch), astragali radix (dried root of *Astragalus membranaceus* (Fisch.) Bge), and black soybean seed coats (seed coats of *Glycine max* (L.) Merr). All four of its components have been extensively studied, and several studies have shown that some of the components in SF can treat RA. For example, a mixture of scolopendra and scorpion powder can attenuate inflammation and articular damage by normalizing T lymphocyte subsets and the balance of Th1/Th2 cytokines [16]. Astragalus alleviated arthritis in rats by regulating the OPG/RANKL/NF-κB pathway [17]. Black soybean seed coats alleviated arthritis in CIA mice by suppressing nuclear factor kappa-B (NF-κB) signaling [18]. Although SF is a traditional prescription for the treatment of RA and show beneficial clinical effects, only limited studies have been conducted on the pharmacological research of SF to date and the mechanism of SF in treating RA is still unclear [15, 19]. In the present study, we investigated the therapeutic potency of SF in a CIA mouse model and explored the possible mechanism of SF in RA treatment via in vivo and in vitro experiments.

## Methods

### Reagents

Processed scorpion (Batch No. 20100108), scolopendra (Batch No. 20200301), astragali radix (Batch No. 2010012), black soybean seed coats (Batch No. 200414), and methotrexate (MTX) were purchased from Jiangsu Integrated Traditional Chinese and Western Medicine Hospital (Nanjing, Jiangsu, China). Calycosin and protocatechuic acid were purchased from Yuanye Bio-Technology (Shanghai, China). Bovine type II collagen and Freund’s adjuvant were purchased from Chondrex (Redmond, WA, USA). An enzyme-linked immunosorbent assay (ELISA) kit for anti-bovine collagen II-specific antibodies was purchased from Chondrex (Redmond, WA, USA). Cell counting kit-8 was purchased from MCE (NJ, USA). Antibodies against IL-17A and retinoic acid receptor-related orphan nuclear receptor γt (RORγt) were purchased from Abcam (Cambridge, UK). Antibodies against STAT3 and phosphorylated STAT3 (pSTAT3, Tyr705) were purchased from Cell Signaling (Boston, USA). Antibodies against IL-6, TNF-α, and interleukin-10 (IL-10) were purchased from Servicebio (Wuhan, China). ELISA kits for TNF-α, IL-6, IL-17A, and IL-10 were purchased from R&D Systems (Minneapolis, MN, USA). Antibodies against anti-mouse CD3, anti-CD4, anti-CD25, anti-Foxp3, and anti-IL-17A were purchased from BD Biosciences (Franklin Lakes, NJ, USA). Anti-mouse CD4 magnetic particles were purchased from BD Biosciences (Franklin Lakes, NJ, USA). The permeabilization solution kit was purchased from BD Biosciences (Franklin Lakes, NJ, USA). The cell stimulation cocktail was purchased from Invitrogen (Carlsbad, CA, USA). The HiScript Reverse Transcription system was purchased from Vazyme Biotech (Nanjing, Jiangsu, China). TRIzol reagent was purchased from Invitrogen (Carlsbad, CA, USA).

### Preparation of SF

SF was provided by the pharmacy of Jiangsu Province Hospital on Integration of Chinese and Western Medicine (Jiangsu, China) and was composed of scolopendra, scorpion, astragali radix and black soybean seed coats. The daily dose of SF crude drug administered by humans in the clinic was 0.85 g/kg. The processed scorpion and scolopendra were crushed through an 80-mesh sieve to form a fine powder, which was further crushed into ultrafine powder using a cryogenic ball mill. Astragali radix and black soybean seed coats were decocted twice with 600 mL of water at 100 °C for 1 h to obtain an aqueous extract, based on a previously reported procedure [20]. The aqueous extract was freeze-dried at − 80 °C for 72 h to obtain a freeze-dried powder. The freeze-dried SF powder was dissolved in double distilled water and used in the in vivo and in vitro experiments.

### HPLC-Q-TOF-MS analysis and quality control of SF

HPLC-Q-TOF-MS analysis was performed to identify the main compounds in SF (50 mg/mL). Chromatographic separation was performed using an Agilent C18 column (3.0 mm × 100 mm, 2.7 μm; Agilent Technologies, Santa Clara, CA, USA) at 40 °C. The mobile phase consisted of water containing 0.1% phosphoric acid (A) and acetonitrile (B). The gradient program was set as follows: 0–0.01 min, 5% B; 0.01–25 min, 5–95% B; 25–27 min, 5% B; 27–30 min, 5% B. The mobile phase flow rate was 0.3 mL/min, and the sample injection volume was 2 µL. Electrospray ionization (ESI) with positive ion modes was used for mass detection. The source parameters were set as follows: spray voltage, 4.5 kV; gas temperature, 550 °C; pressure of nebulizer gas, 55 psi; full scan range, m/z 50–1000.

The main ingredient in SF (50 mg/mL) was confirmed using an Agilent 1260 liquid chromatography system. Methanol (80%) was used as the extraction solvent for the ultrasonic treatment of SF. The SF was separated on an X-Bridge C18 column (250 mm × 4.6 mm, 5 μm) maintained at 25 °C. The mobile phase consisted of acetonitrile (A) and 0.1% phosphate buffer (B) in a gradient elution: 0–10 min, 5% A; 10–11 min, 5–15 % A; 11–20 min, 15–18% A; 20–30 min, 18–20 % A; 31–40 min, 20–30% A; 31–40 min, 30–37 % A; 40–46 min, 37–90 % A. The flow rate was 1 mL/min, the injection volume was 10 µL, and detection was set at 260 nm. Chromatographic data were acquired and analyzed using the Empower software (Agilent). The peaks of protocatechuic acid and calycosin in SF were identified by comparing peak retention times with those of the reference compounds.

### Animals

Six-to-eight-week-old female C57BL/6 and DBA/1J mice were purchased from Changzhou Cavens Experimental Animal Co., Ltd. (Jiangsu, China) and maintained under specific pathogen-free conditions at the Animal Center of Jiangsu Province Academy of Traditional Chinese Medicine. All mice raised in circumstances that alternated between 12 h of light and 12 h of darkness at the temperature of 20–25 °C and relative humidity of 50–70 %. All animal experimental procedures were performed in accordance with the national and international guidelines and regulations, and were approved by the Animal Ethics Committee of Jiangsu Province Academy of Traditional Chinese Medicine.

### CIA induction and drug administration

CIA mice were immunized twice using bovine type II collagen. In the first immunization, bovine collagen II and Freund’s complete adjuvant were mixed and administered as an intradermal injection at the base of the tail in each mouse (100 µg/mouse). On day 21, a booster injection was given using bovine collagen II and Freund’s incomplete adjuvant. DBA/1J female mice were randomly divided into five groups: normal group (Normal, n = 5), CIA vehicle group (CIA vehicle, n = 5), methotrexate group (MTX, n = 5), SF low-dose group (SF-L, 183 mg/kg, n = 5), and SF high-dose group (SF-H, 550 mg/kg, n = 5). The doses of SF and MTX used in this study were determined from the recommended dosages for humans. Though the doses of SF low (183 mg/kg) and SF high (550 mg/kg) were calculated based on a person’s daily administration of 1 or 3 g of scorpion and scolopendra, both SF low and SF high dose treated mice were also administered of 0.99 g/kg black soybean seed coats and 0.66 g/kg astragali radix based on a person’s daily dose (30 g for black soybean seed coats and 20 g for astragali radix). Oral administration of SF (183 or 550 mg/kg/day) began 28 days after the first immunization, and MTX was administered at a dose of 920 mg/kg twice a week. Mice in the normal and vehicle groups were administered an equal volume of deionized water at the same time points.

### Evaluation of arthritis

The incidence of arthritis was evaluated every 3–4 days following immunization. The severity of arthritis was assessed on a scale of 0–4, based on the following previously described criteria [21]: 0 = no evidence of erythema or swelling; 1 = erythema and mild swelling extending to the tarsals or ankle joint; 2 = erythema and mild swelling extending from the ankle to the tarsals; 3 = erythema and moderate swelling extending from the ankle to metatarsal joints; 4 = erythema and severe swelling encompassing the ankle, foot, and digits, or ankylosis of the limb. The arthritis score for each mouse was expressed as the sum of the scores of all four limbs. The highest arthritis score that a mouse could have was 16. Hind paw swelling was measured using a paw volume meter (Woodland Hills, CA, USA).

### Histological evaluation

The mice were sacrificed after 30 days of SF treatment and the hind limbs (including paws and ankles) were collected. The hind limbs were fixed in 4 % paraformaldehyde solution, which was then decalcified with 10 % EDTA for 1 month. After that, the hind limbs were paraffin-embedded, and tissues sectioned and stained with hematoxylin and eosin (H&E), and Safranin O. The degree of histopathological damage was evaluated based on previously described criteria [22].

### Measurement of cytokine and bovine collagen-specific IgG levels

Blood was collected from each mouse on day 30 of treatment and clotted at 25 °C for 1 h. Blood was centrifuged at 4000 rpm for 15 min to obtain serum, which was then stored at − 80 °C until use. The levels of cytokines and bovine collagen-specific IgG were measured using ELISA kits according to the manufacturers’ instructions.

### Immunohistochemistry

The hind limb tissue sections were incubated with anti-IL-6, anti-TNF-α, anti-IL-10, and anti-IL-17A antibodies. The spleen tissue sections were incubated with RORγt, STAT3, and pSTAT3 (Tyr705) antibodies, respectively. After incubation with HRP-conjugated goat anti-rabbit and anti-mouse IgG antibody, the expression of IL-6, TNF-α, IL-10, IL-17A, RORγt, STAT3, and pSTAT3 (Tyr705) were visualized using a DAB kit (Servicebio, Wuhan, China). The quantitative analysis of IL-6, TNF-α, IL-10, IL-17A, RORγt, STAT3, and pSTAT3 (Tyr705) were performed using Image J 1.37v. The results were expressed as the mean region of interest, and the average optical density was used for statistical analysis.

### T-cell isolation and cell viability assay

CD4^+^ T cells were isolated from the splenocytes of 6-to-8-week-old female C57BL/6 mice using anti-mouse CD4 magnetic particles and an IMag Cell Separation Magnet (BD Biosciences, Franklin Lakes, NJ, USA). To evaluate the effect of SF on the viability of CD4^+^ T cells, the cells were treated with different concentrations of SF (0.1–1 mg/mL) for 72 h, and cell viability was detected with a cell counting kit-8.

### In vitro Th17 cell differentiation

The polarization of Th17 cells was performed as previously described [23], CD4^+^ T cells were stimulated with plate-bound anti-CD3 (1 µg/mL), anti-CD28 (1 µg/mL), anti-interleukin-4 (2 µg/mL), anti-interferon-γ (IFN-γ, 2 µg/mL), transforming growth factor-β (TGF-β, 2 ng/mL), IL-6 (30 ng/mL), interleukin-23 (IL-23, 20 ng/mL), and interleukin-1β (IL-1β, 10 ng/mL) for 72 h. After 3 days, the cells were re-stimulated with a cell stimulation cocktail and then stained with anti-mouse CD4 and anti-IL-17A antibodies. The level of IL-17A in the cell supernatant was detected using an ELISA kit.

### Flow cytometry

After oral administration of SF for 30 days, all mice were sacrificed, and the spleen and lymph nodes extracted, mashed and washed with phosphate-buffered saline (PBS). For intracellular IL-17A and Foxp3 staining, the splenocytes and lymph node cells were stimulated with a leukocyte activation cocktail for 5 h. Next, cells were stained with surface FITC-conjugated anti-CD4 antibody or stained with FITC-conjugated anti-CD4 and APC-conjugated anti-CD25 antibodies. After fixation and permeabilization, cells were stained with PE-conjugated anti-Foxp3 or PE-conjugated anti-IL-17A antibodies. Finally, the cells were analyzed via flow cytometry (BD Biosciences, Franklin Lakes, NJ, USA). Flow Jo v10 was used to further analyze the levels of Treg and Th17 cells.

### Western blot

CD4^+^ T cells were cultured for 3 days with or without SF (0.1, 0.5, and 1 mg/mL) under Th17-polarizing conditions. Then, total cellular protein was extracted. Protein samples were separated using 10 % sodium dodecyl sulfate-polyacrylamide gel electrophoresis and transferred to a polyvinylidene fluoride membrane (Merck, Darmstadt, Germany). The membrane was preincubated with 5 % BSA in Tris buffered saline (TBS) for 2 h at room temperature (Sigma, Saint Louis, USA). Diluted STAT-3, pSTAT-3, and GAPDH antibodies (all from Cell Signaling Technology) by 1:1000, and the samples were incubated overnight at 4 °C. After incubation with HRP-conjugated goat anti-rabbit and anti-mouse IgG antibody, the samples were washed with TBST, and the bands were detected by the Odyssey laser imager (LI-COR, Nebraska, USA). Data were analyzed with Image J 1.37v.

### RNA extraction and real-time qPCR

CD4^+^ T cells were cultured for 3 days with or without SF (0.1, 0.5, and 1 mg/mL) under Th17-polarizing conditions. Total RNA was extracted from CD4^+^ T cells using TRIzol reagent according to the manufacturer’s protocol. cDNA was synthesized using the HiScript Reverse Transcription system. A 7500 real-time PCR system (Applied Biosystems, Waltham, MA, USA) was used for PCR amplification. All reactions were performed using the ChamQ Universal SYBR qPCR Master Green kit (Vazyme Biotech, Nanjing, China). The 2^−ΔΔCt^ method was used for data analysis. The primer sequences used are listed in Table 1.

**Table 1:** Primers used for real-time qPCR in the present study

Gene	Forward	Reverse
Mouse RORγt	5′-GACCCACACCTCACAAATTGA-3′	5′-AGTAGGCCACATTACACTGCT-3′
Mouse IL-17A	5′-TTTAACTCCCTTGGCGCAAAA-3′	5′-CTTTCCCTCCGCATTGACAC-3′
Mouse IRF4	5′-TCCGACAGTGGTTGATCGAC-3′	5′-CCTCACGATTGTAGTCCTGCTT-3′
Mouse GAPDH	5′-TGTGGATGGCCCCTCTGGAA-3′	5′-TGACCTTGCCCACAGCCTTG-3′

### Statistical analysis

GraphPad Prism (GraphPad Software version 7.0, San Diego, CA, USA) was used for statistical analysis. One-way ANOVA and two-way ANOVA with Dunnett’s post-hoc multiple comparison tests were used to determine statistical significance. Statistical significance was established at p < 0.05.

## Results

### Identification of chemical compounds in SF by HPLC-Q-TOF-MS and quality control of SF

**Fig. 1:**
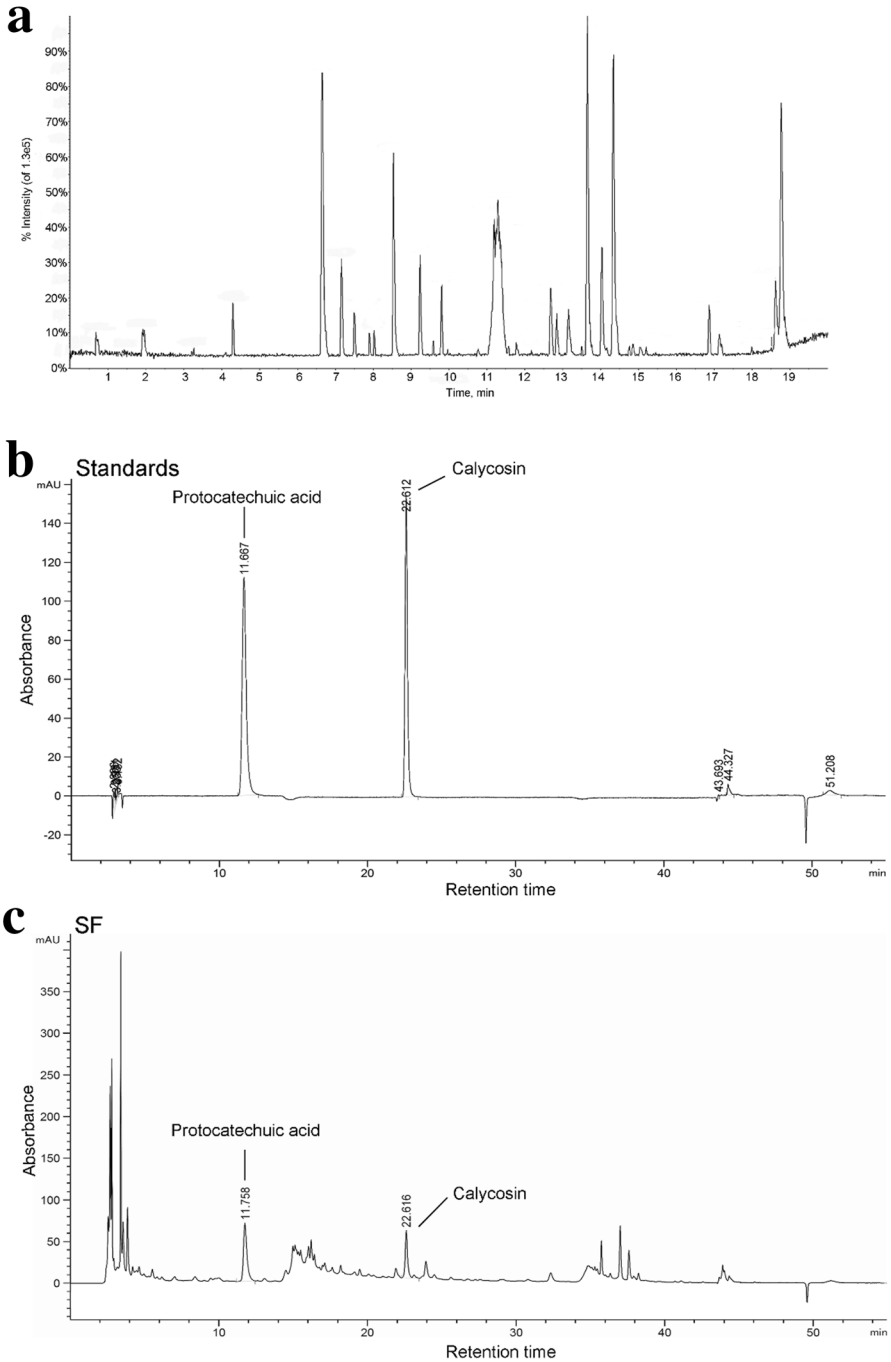
Identification of chemical compounds in SF by HPLC-Q-TOF-MS and quality control of SF. **a** Representative base peak intensity chromatogram of SF based on HPLC-Q-TOF-MS in positive mode. **b** HPLC chromatograms of calycosin and protocatechuic acid at 260 nm. **c** HPLC chromatogram of SF at 260 nm

In the HPLC-Q-TOF-MS experiment, 13 compounds were detected in the SF, as shown in Fig. 1a and Table 2. The 13 compounds were tentatively characterized based on their formula and retention times. According to the Chinese Pharmacopoeia, protocatechuic acid and calycosin were used as references to verify the composition of SF. The representative HPLC chromatograms of standards and SF are shown in Fig. 1b, c. The content of protocatechuic acid and calycosin in SF were analyzed and generated regression equations using the peak area and concentrations. The content of protocatechuic acid in SF was 0.553 mg/g. The content of calycosin in SF was 0.291 mg/g.

**Table 2:** Characteristics of compounds identified in SF through HPLC-Q-TOF-MS

NO	Rt (min)	Identification	Formula	m/z	Error (ppm)
1	0.66	L (+)-Arginine	C_6_H_14_N_4_O_2_	[M+H]^+^ 175.1189	− 0.6
2	0.72	L-Proline	C_5_H_9_NO_2_	[M+H]^+^ 116.0706	− 0.5
3	3.22	2-(7,8-dimethyl-2,4-dioxobenzo[g]pteridin-10-yl) acetaldehyde	C_14_H_12_N_4_O_3_	[M+NA]^+^ 307.0801	4.7
4	4.08	Epicatechin	C_15_H_14_O_6_	[M+H]^+^ 291.0863	2
5	6.71	L-Leucine	C_6_H_13_NO_2_	[M+COOH]^+^ 176.0917	2
6	8.11	Calycosin	C_16_H_12_O_5_	[M+H]^+^ 285.0757	0.8
7	8.57	Fmoc-L-1,2,3,4-tetrahydro-norharman-3-carboxylic acid	C_27_H_22_N_2_O_4_	[M+H]^+^ 439.1652	− 5
8	11.57	Palmitic acid	C_16_H_32_O_2_	[M+NH_2_]^+^ 272.2584	3.1
9	11.83	Oleic acid	C_18_H_34_O_2_	[M+NH_2_]^+^ 298.2740	0.4
10	12.84	Ethyl 3,4-dihydroxybenzoate	C_9_H_10_O_4_	[M+H]^+^ 183.0651	1.9
11	13.73	Astragaloside I	C_45_H_72_O_16_	[M+COOH] 913.4791	3.2
12	14.39	Delphinidin chloride	C_15_H_11_CLO_7_	[M+COOH]^+^ 383.0164	0.8
13	15.39	α-Linolenic acid	C_18_H_32_O_2_	[M+H]^+^ 279.2318	1.7

### SF suppressed the development of arthritis in CIA mice

**Fig. 2:**
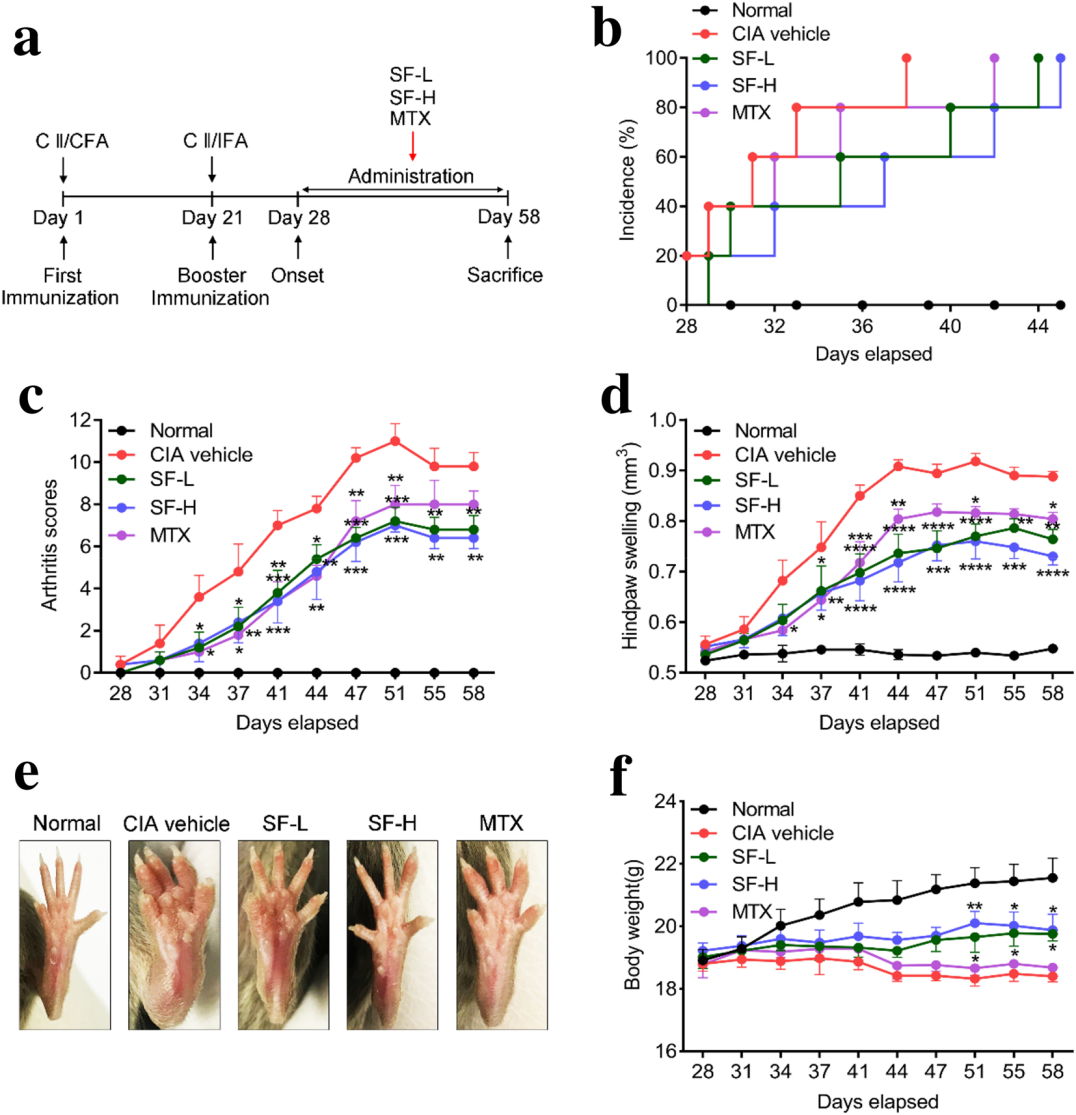
Effect of SF on onset and clinical symptoms of arthritis in CIA mice. **a** Flow chart of CIA model induction and treatment options. **b** Incidence of arthritis in each group (n = 5). **c** Arthritis scores of mice in each group (n = 5). **d** Hind paw swelling of mice in each group (n = 5). **e** Representative images of feet in each group after one-month administration (n = 5). **f** Body weight of mice in each group. Values are presented as means ± SEM. Two-way ANOVA and post hoc Dunnett’s test were performed between multiple groups. *p < 0.05, **p < 0.01, ***p < 0.001, ****p < 0.0001 compared with CIA vehicle group

DBA/1J mice were immunized with bovine collagen II twice, and SF was administered orally every day, starting 28 days after the first immunization (Fig. 2a). Mice began to show symptoms of arthritis 28 days following the first immunization. As shown in Fig. 2b, the incidence of arthritis reached 100% 38, 42, 44, and 45 days after immunization in the CIA vehicle group, the MTX group, the SF low-dose group, and the SF high-dose group, respectively. Arthritis scores were assessed and swelling of the hind paw was measured after the booster immunization. Compared with mice in the CIA vehicle group, the arthritis scores of mice in the SF low-dose and SF high-dose groups were significantly reduced from the days 37 to 58 following the first immunization (Fig. 2c). And the arthritis scores of mice in the MTX group was also significantly reduced from the days 34 to 51. Moreover, compared with mice in the CIA vehicle group, the hind paw swelling of mice in the SF (low-dose and high-dose) and MTX groups decreased significantly after day 37 of immunization (Fig. 2d). After administration of SF for 30 days, the hind paw of mice in the CIA vehicle group were severely swollen and deformed, whereas no significant deformation was found among the SF (low-dose and high-dose) and MTX groups (Fig. 2e). Additionally, the body weights of mice in the SF low-dose and SF high-dose groups on days 51, 55 and 58 after immunization were significantly higher than those of the CIA vehicle group mice (Fig. 2f).

### SF reduced ankle joint bone destruction and serum levels of CII specific IgG

**Fig. 3:**
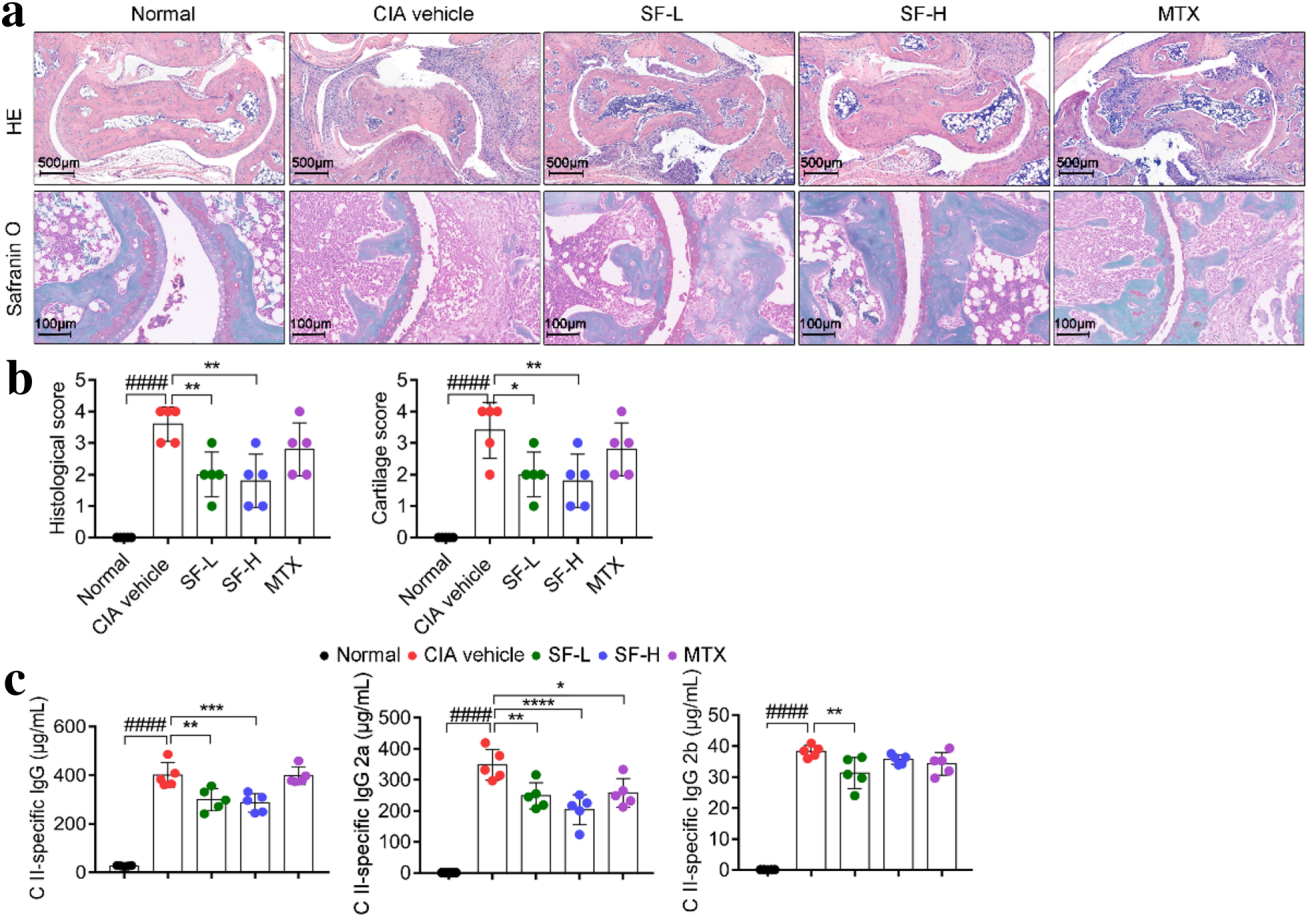
Effect of SF on joint bone destruction and serum bovine CII-specific IgG level in CIA mice. **a** Representative image of H&E and Safranin O staining in ankle joints (Scale bars, 500 and 100 μm). **b** Evaluation of joint pathology and cartilage of mice in each group (n = 5). **c** Levels of total anti-bovine collagen II IgG, IgG2a, and IgG2b in mice sera (n = 5). One-way ANOVA and post hoc Dunnett’s test were performed between multiple groups. ^####^p < 0.0001, compared with Normal group; *p < 0.05, **p < 0.01, ***p < 0.001, ****p < 0.0001, compared with CIA vehicle group

Assessment of joint pathology and cartilage destruction by H&E and Safranin O staining showed that the hind paw joints of CIA mice treated with SF exhibited a significant reduction in cartilage loss and joint destruction compared with the CIA vehicle mice (Fig. 3a). The joint histopathological and cartilage scores of mice in the SF low-dose and SF high-dose groups were also significantly lower than those in the CIA vehicle group (Fig. 3b). Moreover, the levels of bovine collagen II-specific IgG and IgG2a in the sera of mice in the SF low-dose and SF high-dose groups were also significantly lower than those in the CIA vehicle group (Fig. 3c). However, the levels of IgG2b in the sera of mice in the SF low-dose group were lower than those in the CIA vehicle group and the levels of IgG2a in the sera of mice in the MTX group were lower than those in the CIA vehicle group (Fig. 3c).

### SF decreased the levels of IL-6, TNF-α, and IL-17A, and increased the level of IL-10 both in the serum and joints of CIA mice

**Fig. 4:**
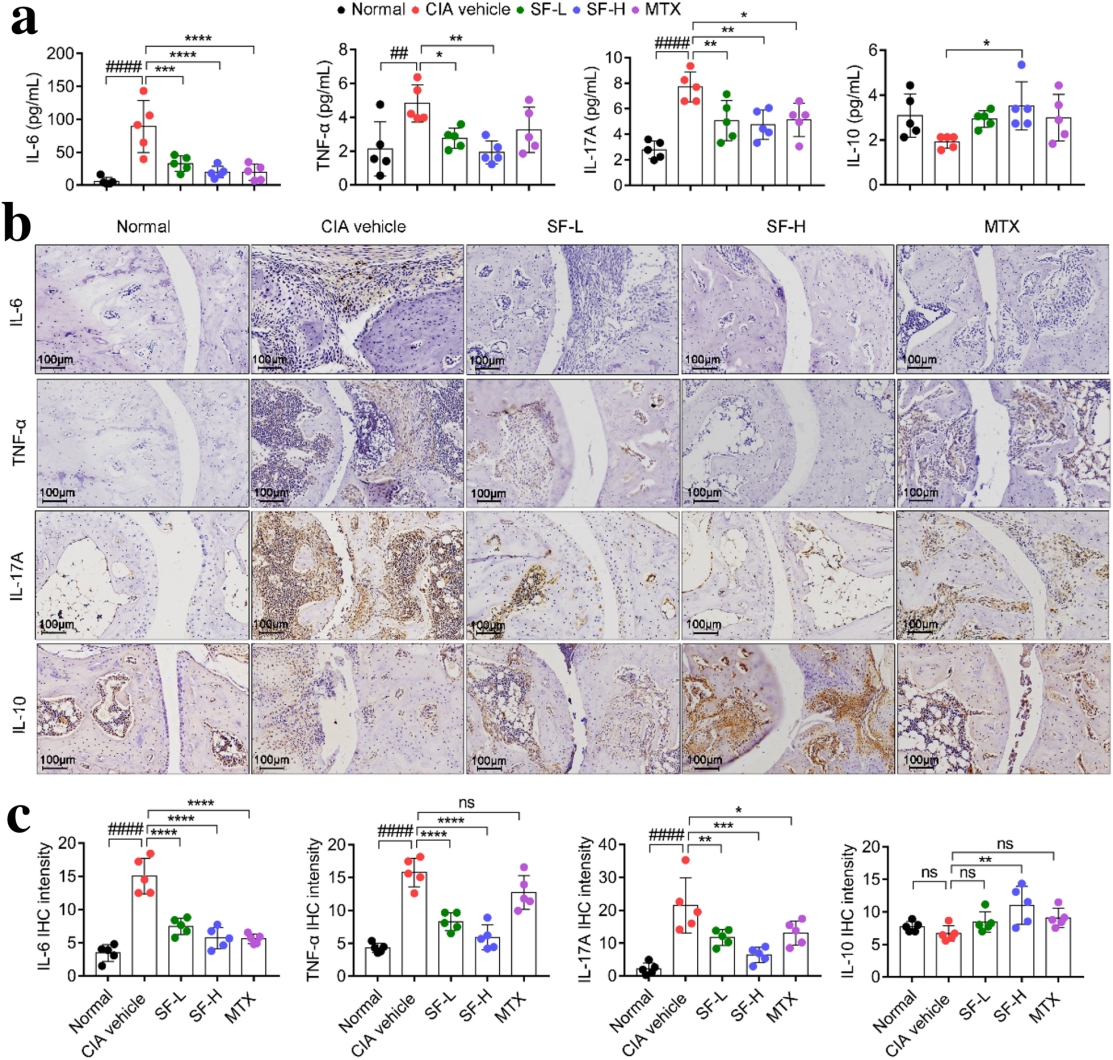
Effect of SF on inflammatory cytokines in serum and joints of CIA mice. **a** Levels of TNF-α, IL-6, IL-17A, and IL-10 in mice sera (n = 5). **b** Representative images of immunohistochemical staining of TNF-α, IL-6, IL-17A, and IL-10 in ankle joints (Scale bars, 100 μm). **c** Levels of TNF-α, IL-6, IL-17A, and IL-10 in mice ankle joints (n = 5). One-way ANOVA and post hoc Dunnett’s test were performed between multiple groups. ^##^p < 0.01, ^####^p < 0.0001, compared with Normal group; *p < 0.05, **p < 0.01, ***p < 0.001, ****p < 0.0001, compared with CIA vehicle group

Next, we measured the inflammatory cytokines associated with RA. Compared with the CIA vehicle group, the sera levels of proinflammatory cytokines TNF-α, IL-6, and IL-17A were significantly decreased in the SF low-dose and SF high-dose groups (Fig. 4a), and the levels of IL-6 and IL-17A were significantly decreased in the MTX group compared to those in the CIA vehicle group (Fig. 4a). Moreover, the levels of IL-10 in the sera of mice in the SF high-dose group were higher than those in the CIA vehicle group (Fig. 4a). In addition, the expression levels of TNF-α, IL-6, and IL-17A were significantly downregulated in the ankle joints of the SF (low-dose and high-dose) compared with those in the CIA vehicle group (Fig. 4b, c). Meanwhile, the expression level of IL-10 was significantly upregulated in the ankle joints of the SF high group compared with those in the CIA vehicle group (Fig. 4b, c).

### SF treatment rebalanced Th17/Treg ratio in the spleen and lymph nodes of CIA mice

**Fig. 5:**
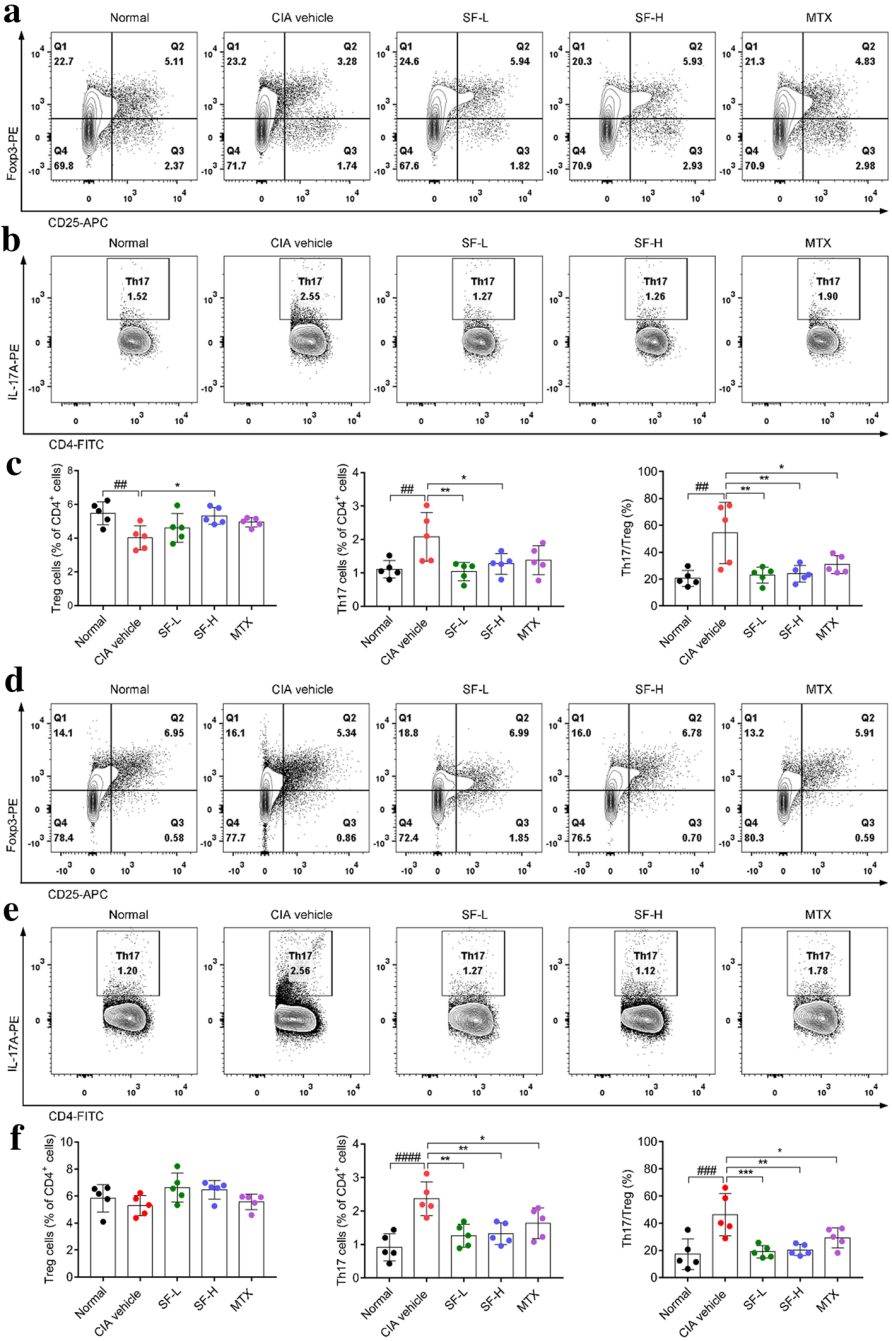
Effect of SF on Treg and Th17 cells in the spleen and lymph nodes of CIA mice. **a** Representative image of CD4^+^CD25^+^Foxp3^+^ cells in spleen after one-month administration. **b** Representative image of CD4^+^IL-17A^+^ cells in spleen after one-month administration. **c** Proportion of Treg and Th17 cells and ratio of Treg/Th17 in the spleen of each group of mice (n = 5). **d** Representative image of CD4^+^CD25^+^Foxp3^+^ cells in lymph nodes after 1-month administration. **e** Representative image of CD4^+^IL-17A^+^ cells in lymph nodes after 1-month administration. **f** Proportion of Treg and Th17 cells and ratio of Treg/Th17 in the lymph nodes of each group of mice (n = 5). One-way ANOVA and post hoc Dunnett’s test were performed between multiple groups. ^##^p < 0.01, ^###^p < 0.001, ^####^p < 0.0001, compared with Normal group; *p < 0.05, **p < 0.01, ***p < 0.001, compared with CIA vehicle group

As shown in the above results, the sera and joints levels of TNF-α, IL-6, and IL-17A of the SF treatment groups were significantly lower than those in the CIA group, while sera and joints level of IL-10 was significantly increased. We next investigated the effect of SF on the immune balance in CIA mice. After administration of SF for 30 days, we measured the numbers of Treg and Th17 cells in the spleen and lymph nodes of the mice. As shown in Fig. 5, compared to the normal mice, the number of Th17 (CD4^+^IL-17^+^) cells was significantly increased in the spleen and lymph nodes of CIA mice, while the number of Treg (CD4^+^CD25^+^Foxp3^+^) cells was significantly decreased in the spleen of CIA mice. Meanwhile, the ratio of Th17/Treg cells in the spleen and lymph nodes of the CIA vehicle group was significantly higher than that in the normal group (Fig. 5c, f). However, compared with mice in the CIA vehicle group, the numbers of Th17 cells and the ratio of Th17/Treg in the spleen and lymph nodes of the SF (low-dose and high-dose) and MTX groups were significantly reduced (Fig. 5c, f). Note that only the number of Treg cells in the spleen of mice in the SF high-dose group increased significantly and the number of Th17 cells in the lymph nodes of mice in the MTX group decreased significantly (Fig. 5a, e). In addition, we also detected the levels of Th1 and Th2 in the spleen of each group of mice. Although the Th1 cell level of the CIA group was significantly higher than that of the normal group, and the Th1/Th2 ratio of the CIA group was also significantly lower than that of the normal group, there was no significant difference in Th1, Th2 and Th1/Th2 between the SF-L and SF-H groups and the CIA vehicle group (Additional file 1: Fig. 1).

### SF decreased the expression levels of RORγt and pSTAT3 in the spleen of CIA mice

**Fig. 6:**
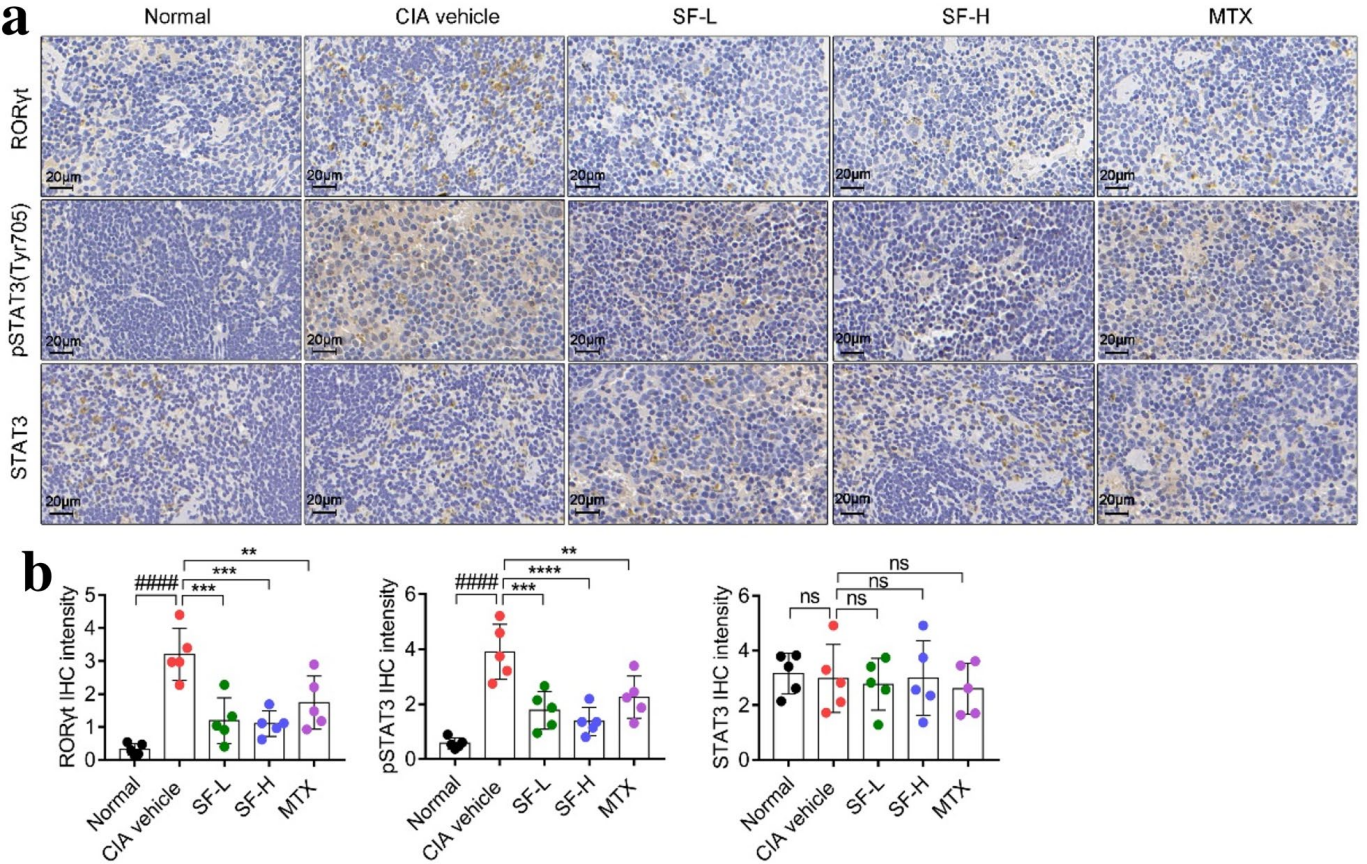
Effect of SF on the levels of RORγt, STAT3, and pSTAT3 in the spleen of CIA mice. **a** Representative image of immunohistochemical staining of RORγt, STAT3, and pSTAT3 (Tyr705) in the spleen (Scale bars, 20 μm). **b** Levels of RORγt, STAT3, and pSTAT3 (Tyr705) in the spleen (n = 5). One-way ANOVA and post hoc Dunnett’s test were performed between multiple groups. ^####^p < 0.0001, compared with Normal group; **p < 0.01, ***p < 0.001, ****p < 0.0001, compared with CIA vehicle group

To investigate the signaling pathway that SF reduced the level of Th17 cells in CIA mice, the expression levels of RORγt, STAT3, and pSTAT3 (Tyr705) in the spleen of each group of mice were measured. As shown in Fig. 6a, b, compared with the normal group, the expression levels of RORγt and pSTAT3 (Tyr705) in the spleen of the CIA vehicle group increased significantly. However, compared with the CIA vehicle group, the expression levels of RORγt and pSTAT3 (Tyr705) in the spleen of the SF treatment group and the MTX group were significantly reduced.

### SF inhibited Th17 cell differentiation and Th17-related mRNA expression in vitro

**Fig. 7:**
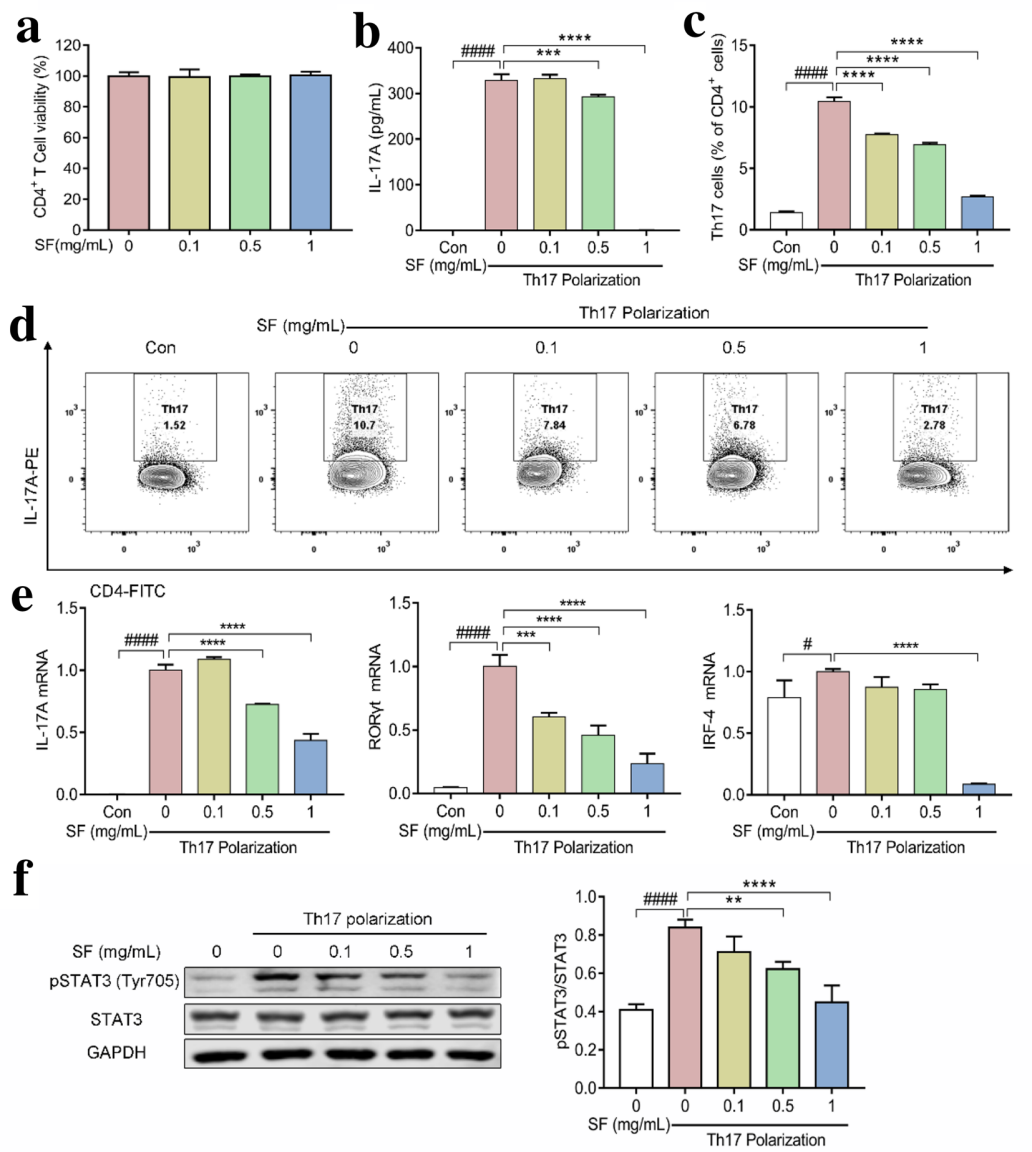
Effects of SF on Th17 differentiation in vitro. **a** The effect of SF (0.1–1 mg/mL) on CD4^+^ cell viability. **b** IL-17A levels in the cell supernatant under Th17-polarizing conditions (n = 3). **c** Proportion of Th17 cells in each group from three independent experiments (n = 3). **d** Representative images of CD4^+^IL-17A^+^ cells under Th17-polarizing conditions. **e** The mRNA expression levels of the Th17 cell-associated genes IL-17A, RORγt and IRF4 (n = 3). **f** The levels of STAT3 and pSTAT3 (Tyr705) in each group under Th17-polarizing conditions (n = 3). One-way ANOVA and post hoc Dunnett’s test were performed between multiple groups. ^#^p < 0.05, ^####^p < 0.0001, compared with unpolarized group; **p < 0.01, ***p < 0.001, ****p < 0.0001, compared with polarized group without treatment

Next, we investigated the effect of SF on Th17 cells using an in vitro model of Th17 cell differentiation. First of all, CD4^+^ T cells were isolated from the splenocytes of C57BL/6 mice and treated with different concentrations of SF (0.1–1 mg/mL). As shown in Fig. 7a, SF (0.1–1 mg/mL) had no effect on CD4^+^ T cell viability. The CD4^+^ T cells were then incubated under Th17-polarizing conditions in the presence or absence of SF (0.1–1 mg/mL). SF reduced IL-17A levels in the cell supernatant in a dose-dependent manner (Fig. 7b). Moreover, compared with unpolarized CD4^+^ T cells, the number of Th17 cells increased significantly after polarization, while Th17 cells in the SF treatment group decreased significantly in a dose-dependent manner (Fig. 7c, d). Next, the mRNA expression of Th17-associated cytokines and Th17-specific transcription cytokine RORγt were analyzed. Compared with unpolarized CD4^+^ T cells, the mRNA levels of IL-17A, RORγt, and IRF4 in CD4^+^ T cells under Th17 polarization conditions increased significantly, while the mRNA levels of IL-17A and RORγt in the SF treatment group decreased significantly in a dose-dependent manner (Fig. 7e). However, only SF 1 mg/mL significantly reduced the mRNA level of IRF4 (Fig. 7e). To investigate the signaling pathway of SF regulating Th17 cell differentiation in vitro, the expression levels of STAT3 and pSTAT3 (Tyr705) in CD4^+^ T cells were evaluated by Western blotting. As shown in Fig. 7f, the level of pSTAT3 (Tyr705) in CD4^+^ T cells was decreased by treatment with SF under Th17 polarization conditions.

## Discussion

Previous studies have shown that traditional Chinese medicine is an alternative medicine for treating long-term chronic diseases such as RA [24]. Traditional Chinese medicine, characterized by monarch, minister, adjuvant and assistant, emphasizes the integrated treatment based on multicomponent action instead of the single-component therapy, which is the typical superiority of traditional Chinese medicine prescription. Treatment by the combination formulae has been verified scientifically for treatment on several diseases as an effective complementary or alternative therapy [25]. In the present study, we demonstrated that SF had a significant therapeutic effect in a CIA mouse model, as evidenced by significantly decreased arthritis scores, joint swelling, and cartilage loss in CIA mice treated with SF compared with CIA vehicle mice. This beneficial effect of SF may be through maintaining the balance of Treg/Th17 cells, down-regulating the expression of TNF-α, IL-6, IL-17A, up-regulating IL-10 expression, and inhibiting the formation of IgG antibodies. Although the main chemical components of SF for the treatment of arthritis have not been identified, it has been reported that peptides in scorpion and scolopendra can reduce inflammation and joint damage by regulating T lymphocytes, and total flavonoids in astragalus can relieve arthritis inflammation in rats, and black soybean coat extract can alleviate joint inflammation in CIA mice by inhibiting Th17 cells [16–18]. The components in SF were complex, and the main components of SF in the treatment of RA need to be further explored.

The pathogenesis of RA is complex, TNF-α and IL-6 are considered to be central hubs in the synovial cytokine network of RA [26]. These cytokines stimulate osteoclast formation and the subsequent degradation of bone and cartilage, and also potently induce the release of other pro-inflammatory mediators, such as IL-1 and granulocyte-macrophage colony-stimulating factor [27]. In the present study, we found that SF significantly decreased the levels of TNF-α, IL-6, and IL-17A in the sera of CIA mice, while sera IL-10 levels were significantly increased. IL-6 mediates Th17 differentiation, and Th17 cells produce inflammatory cytokines, such as IL-17, to augment autoimmune arthritis [9, 28]. Moreover, anti-inflammatory IL-10 endows Treg cells with the ability to suppress pathogenic Th17 cell responses [29]. We also found that the expression of IL-6, TNF-α, and IL-17A in the joints of CIA mice after SF treatment were significantly downregulated, and the expression of IL-10 in the joints of CIA mice after SF treatment was significantly upregulated. Based on these results, we investigated the effect of SF on Treg and Th17 cells in the spleen and lymph nodes of CIA mice.

CD4^+^ T helper (Th) cells play central roles in RA regulation [30]. Naive precursor CD4^+^ T cells develop into Th17 cells when exposed to proinflammatory cytokines, including TGF-β, IL-6, IL-1β, and IL-23 [31], and the pro-inflammatory cytokines produced by Th17 cells are involved in the development of RA [32]. In contrast to Th17 cells, Tregs are indispensable mediators that sustain immune tolerance to self-antigens and help to maintain immune homeostasis [33]. In the present study, although only SF high-dose significantly increased the number of Treg cells in the spleen of CIA mice, SF had a significant inhibitory effect on Th17 cells in the spleen and lymph nodes of CIA mice. The increased of Treg cells in the spleen of CIA mice treated with SF-H may be related to the proliferation and differentiation of naïve CD4 T cells, and further investigation is needed. Furthermore, the ratio of Th17/Treg in the spleen and lymph nodes of CIA mice after SF treatment returned levels similar to that in normal mice. Our findings are consistent with those of other studies showing that maintaining the balance between Th17 and Treg cells is essential for the treatment of RA [6, 8]. Although previous studies have found that a mixture of scolopendra and scorpion powder can attenuate inflammation and articular damage by normalizing T lymphocyte subsets and the balance of Th1/Th2 cytokines [16], our results showed that SF has no significant effect on Th1 and Th2 cells in the spleen of CIA mice.

Although IL-17 antibodies show marked clinical efficacy in psoriasis, targeting IL-17 alone is not sufficient to improve clinical end points in other autoimmune conditions, namely RA [34]. Targeting the Th17 cell lineage may show better anti-RA effects. We found that SF not only significantly decreased the number of Th17 cells in the spleen and lymph nodes of CIA mice, but it could also significantly inhibit the production of Th17 cells in vitro. RORγt is a key transcription factor that controls the differentiation of Th17 cells and induces the expression of genes encoding IL-17 and IL-17 F cytokines [35]. IL-6 can induce the upregulation of RORγt expression, initiating the RORγt signal transduction pathway to promote Th17 cell differentiation [36]. The promotion of Th17 differentiation by RORγt is mainly related to the phosphorylation of STAT3 [37]. Our results showed that SF can significantly decrease the expression levels of RORγt and pSTAT3 (Tyr705) in the spleen of CIA mice, and SF can also inhibit the expression levels of RORγt and pSTAT3 (Tyr705) in vitro. Furthermore, the expression of interferon-regulatory cytokine 4 (IRF4) was also suppressed by SF. IRF4 is essential for the development of Th17 cells and mainly regulates the secretion of IL-17 and IL-21 [38]. These results suggest that SF inhibit the differentiation of Th17 cells by suppressing the expression of pSTAT3 (Tyr705) and RORγt.

This study confirmed, for the first time, that SF could maintain immune balance by inhibiting Th17 cell differentiation, which indicates that SF is an alternative drug candidate for the treatment of RA.

## Conclusions

In conclusion, our results suggest that SF exhibits anti-arthritic effects and restores Th17/Treg homeostasis in CIA mice by inhibiting Th17 cell differentiation.

## Supplementary Information

**Additional file 1.** Effect of SF on Th1 and Th2 cells in the spleen of CIA mice.

## Data Availability

The data used to support the findings of this study are available from the corresponding author upon request.

## Abbreviations

RA: Rheumatoid arthritis
CIA: Collagen-induced arthritis
SF: Soufeng sanjie formula
MTX: Methotrexate
HPLC: High performance liquid chromatography
HPLC-Q-TOF-MS: High performance liquid chromatography-quadrupole-time-of-flight mass spectrometry
TNF-α: Tumor necrosis factor-α
IL-6: Interleukin-6
IL-17: Interleukin-17
IL-10: Interleukin-10
ELISA: Enzyme-linked immunosorbent assay
IRF4: Interferon regulatory factor 4
RORγt: Retinoid-related orphan nuclear receptor γt
pSTAT3 (Tyr705): Phosphorylated STAT3 (Tyr705)
