# Supplementary material for: Soufeng sanjie formula alleviates collagen-induced arthritis in mice by inhibiting Th17 cell differentiation
Source: Chin Med. 2021 May 13;16:39. doi: 10.1186/s13020-021-00448-9 (PMC8117632; doi:10.1186/s13020-021-00448-9)
Supplement: Supplementary file 1 — Additional file 1. Effect of SF on Th1 and Th2 cells in the spleen of CIA mice. [file 13020_2021_448_MOESM1_ESM.docx]

**Additional data**

**1. Effect of SF on Th1 and Th2 cells in the spleen of CIA mice.**

After oral administration of SF for 30 d, all mice were sacrificed, and the spleen extracted, mashed and washed with phosphate-buffered saline (PBS). For intracellular IFN-γ and IL-4 staining (all from Invitrogen), the splenocytes were stimulated with a leukocyte activation cocktail for 5 h. Next, cells were stained with surface FITC-conjugated anti-CD4 antibody. After fixation and permeabilization, cells were stained with PE-cy7–conjugated anti–IFN-γ or APC–conjugated anti-IL-4 antibodies. Finally, the cells were analyzed via flow cytometry (BD Biosciences, Franklin Lakes, NJ, USA). Flow Jo v10 was used to further analyze the levels of Treg and Th17 cells.


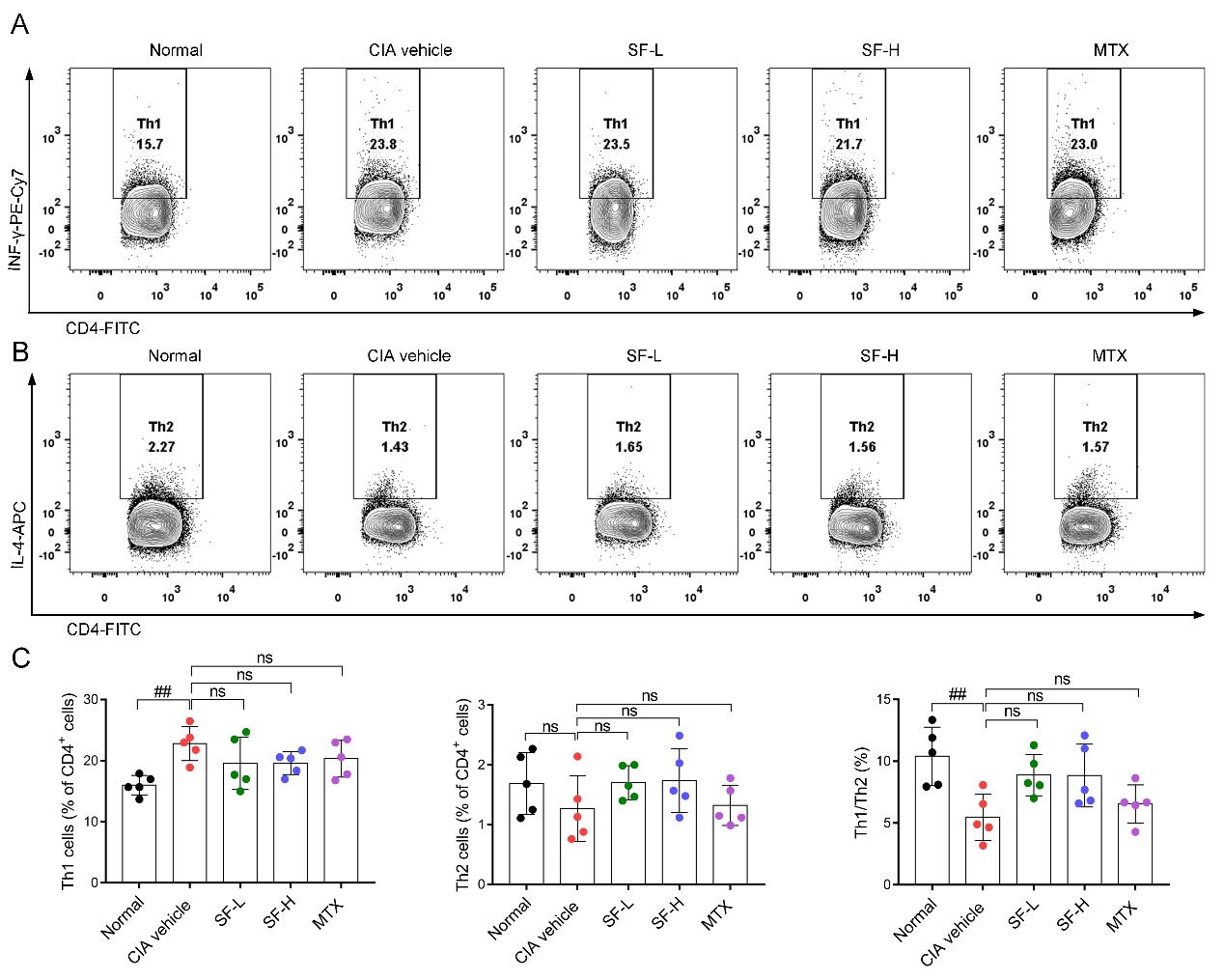


**Figure S1** **Effect of SF on Th1 and Th2 cells in the spleen of CIA mice.** **A** Representative image of Th1 cell in spleen after one-month administration. **B** Representative image of Th2 cell in spleen after one-month administration. **C** Proportion of Th1 and Th2 cells and ratio of Th1/Th2 in the spleen of each group of mice (n=5). One-way ANOVA and post hoc Dunnett’s test were performed between multiple groups. ^##^p<0.01, compared with Normal group.
